# Community successional patterns and inter-kingdom interactions during granular biofilm development

**DOI:** 10.1038/s41522-024-00581-x

**Published:** 2024-10-20

**Authors:** Miguel de Celis, Oskar Modin, Lucía Arregui, Frank Persson, Antonio Santos, Ignacio Belda, Britt-Marie Wilén, Raquel Liébana

**Affiliations:** 1 Department of Genetics, Physiology and Microbiology, Microbiology Unit, Faculty of Biological Sciences, Complutense University of Madrid, Madrid, Spain; 2 Instituto de Ciencias Agrarias; Consejo Superior de Investigaciones Científicas, Madrid, Spain; 3 Division of Water Environment Technology, Department of Architecture and Civil Engineering, Chalmers University of Technology, Gothenburg, Sweden; 4 Present Address: AZTI, Marine Research Division, Basque Research Technology Alliance (BRTA), Sukarrieta, Spain

**Keywords:** Biofilms, Applied microbiology

## Abstract

Aerobic granular sludge is a compact and efficient biofilm process used for wastewater treatment which has received much attention and is currently being implemented worldwide. The microbial associations and their ecological implications occurring during granule development, especially those involving inter-kingdom interactions, are poorly understood. In this work, we monitored the prokaryote and eukaryote community composition and structure during the granulation of activated sludge for 343 days in a sequencing batch reactor (SBR) and investigated the influence of abiotic and biotic factors on the granule development. Sludge granulation was accomplished with low-wash-out dynamics at long settling times, allowing for the microbial communities to adapt to the SBR environmental conditions. The sludge granulation and associated changes in microbial community structure could be divided into three stages: floccular, intermediate, and granular. The eukaryotic and prokaryotic communities showed parallel successional dynamics, with three main sub-communities identified for each kingdom, dominating in each stage of sludge granulation. Although inter-kingdom interactions were shown to affect community succession during the whole experiment, during granule development random factors like the availability of settlement sites or drift acquired increasing importance. The prokaryotic community was more affected by deterministic factors, including reactor conditions, while the eukaryotic community was to a larger extent shaped by biotic interactions (including inter-kingdom interactions) and stochasticity.

## Introduction

Aerobic granular sludge is a biofilm-based process for wastewater treatment that has received much attention in recent years. This technology displays several advantages compared to the activated sludge process, achieving advanced nutrient removal in plants requiring less space and a lower energy demand^1,2^. Aerobic granules are generally developed from activated sludge in sequencing batch reactors (SBRs), where aggregates with high microbial density and diversity are obtained^3^. Substrate and oxygen gradients within the biofilm matrix allow the coexistence of ammonia-oxidizing bacteria (AOB), nitrite-oxidizing bacteria (NOB), denitrifying bacteria and phosphorous accumulating organisms (PAO), thus synchronizing nitrification, denitrification and biological phosphorus removal while degrading the organic carbon^4–7^.

Granulation is a response to specific selection pressures applied in the reactors; however, the underlying mechanisms are still poorly understood. Granules are generally obtained by (1) applying high hydrodynamic shear forces; (2) feast-famine alternation; and (3) washing-out of the non-granulated biomass^4,5,7^. In such reactor conditions, upon switching from planktonic to aggregated mode of growth, microbial populations ensure their persistence in flowing environments that develop under shear forces^8^. Additionally, the applied high shear forces in the reactor, together with the feast-famine alternation and anaerobic feeding strategies applied in SBRs, increases the overall hydrophobicity of the biomass and accelerates microbial aggregation^4,9–11^.

Eukaryotic members of the community play important roles in wastewater treatment contributing to sludge sedimentation and predation upon planktonic bacteria^12–15^, yet few studies have been conducted on their role in the granular sludge process. Filamentous fungi and stalked protists have been proposed to participate in granule development by acting as a backbone for granules, thus increasing the surface to which bacteria can attach^16,17^. Protistan grazing can promote aggregation of wastewater bacteria^18^, since phenotypes can switch towards biofilm development as a survival strategy^19,20^. But protistan grazing can also cause a reduction of bacterial biofilm thickness^21^ and even extend to deep biofilm layers^22^.

The physical factors involved in granule cultivation in SBRs have been extensively studied^4,23–26^ and, although to a lesser extent, so have the microbial dynamics^27–30^. However, the microbial associations and their ecological implications during granular biofilm development are less understood, especially those involving inter-kingdom interactions. Here, we monitored the prokaryotic and eukaryotic community structure and dynamics during the granulation of sludge for 343 days in an SBR, aiming to elucidate the influence of abiotic and biotic factors in granular biofilm development, including their inter-kingdom interactions. For this, we studied the reactor performance and the succession of prokaryotic and eukaryotic community fractions by means of diversity and network analysis, together with null models.

## Results

### Different stages identified during granulation

**Fig. 1:**
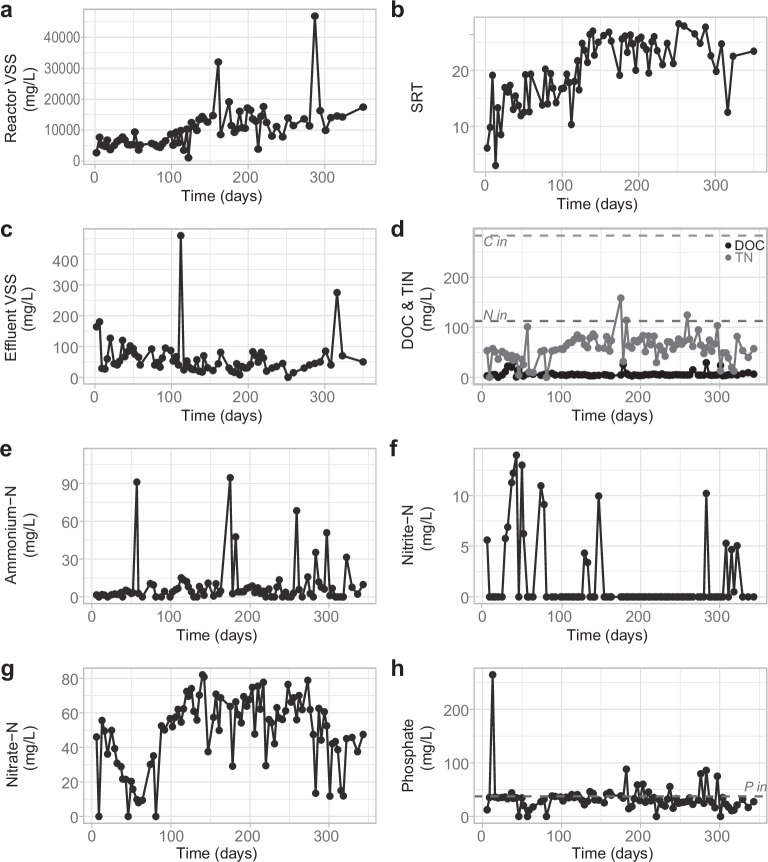
Sludge and performance parameters during the reactor run. **a** reactor volatile suspended solids (VSS) concentrations; **b** sludge retention time (SRT); **c**, effluent volatile suspended solids concentrations; **d** in black, effluent total organic carbon (TOC) and in grey, total inorganic nitrogen (TIN) expressed as the addition of ammonium, nitrite, and nitrate; **e** effluent ammonium concentration; **f** effluent nitrite concentration; **g** effluent nitrate concentration; **h** effluent phosphate concentration. Horizontal dashed lines indicate the influent concentration: total organic carbon 283 mg L^−1^ (C in), total nitrogen 112 mg L^−1^ (N in), and phosphorous 37.6 mg L^−1^ (P in).

Granulation was observed at a long reactor settling time of 30 minutes. Granules started to emerge at day 16 and the mean particle size increased, especially after day 115, once the granules were completely developed (Supplementary Fig. 1). Based on microscopical observations, we divided the granulation process into three stages: floccular stage (days 0–15), intermediate stage (days 16–115) and granular stage (days 116–343). The sludge concentration, with a volatile fraction (microbial fraction) of 77% (SD = 12), and the sludge retention time increased as particle size did, especially in the granular stage (Fig. 1a, b), while the effluent suspended solids concentration was generally below 50 mg L^−1^ (Fig. 1c). Carbon removal was stable during the experiment (Fig. 1d), with dissolved organic carbon (DOC) removal generally above 97%. Complete ammonium removal was achieved during most of the experiment (Fig. 1e), showing a median removal of 97% (SD = 15). Nitrification occurred in the reactor as nitrite was mostly not present in the effluent and nitrate was formed (Fig. 1f, g), especially once granules emerged. Total nitrogen removal was variable along the experiment (48% median, SD = 23, Fig. 1d). Denitrification took place in the reactor as the depletion of nitrate within the SBR cycle was observed (Supplementary Fig. 2). Biological phosphorus removal occurred in the reactor, but the removal was variable and had an increasing trend with time (Fig. 1h).

### Pronounced shift of dominant taxa during the early stages of granulation

**Fig. 2:**
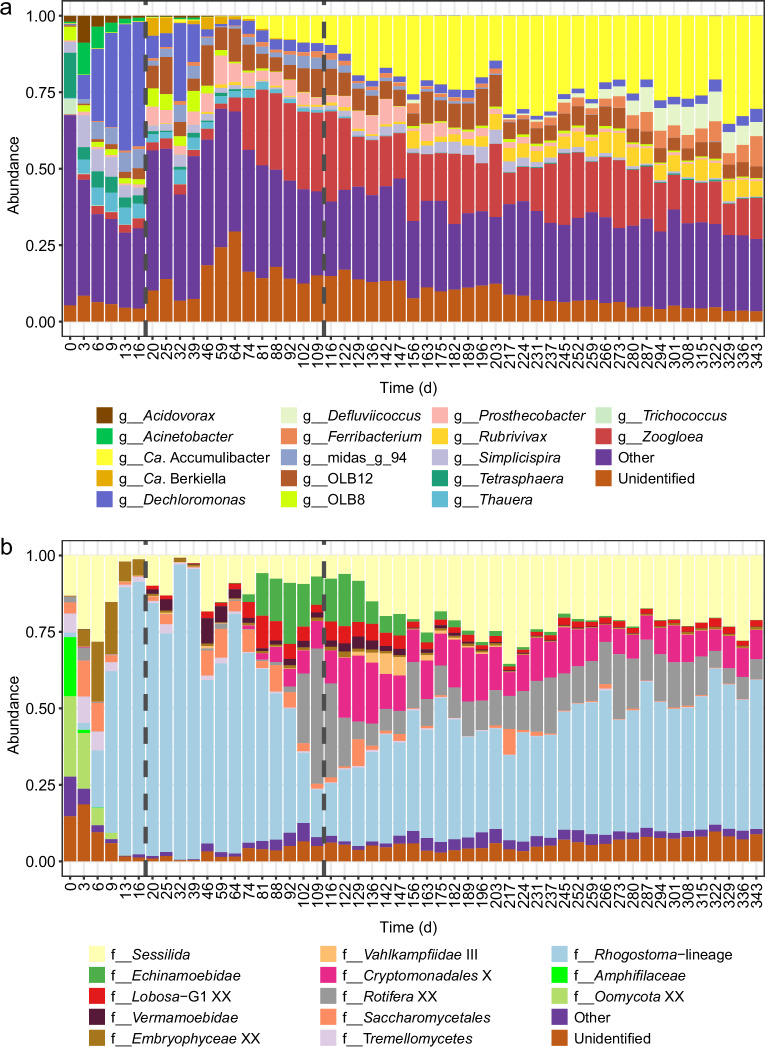
Temporal dynamics of microbial taxonomic composition. Taxonomic distribution of **a** prokaryotic community at the genus-level and **b** eukaryotic community at the family-level abundances. “Other”, includes minor prokaryotic genera and eukaryotic families; and “Unidentified”, taxonomically unassigned taxa.

Overall, we observed marked compositional changes during the floccular and intermediate stages while the granular stage was characterized by being more compositionally stable in both eukaryotic and prokaryotic community fractions. The microbial community composition displayed drastic shifts after the reactor start-up (Fig. 2). The initial prokaryotic community was more complex than at subsequent timepoints. The genera *Acinetobacter*, *Thauera* and *Dechloromonas* had high relative abundance and the latter increased during the floccular stage. During the intermediate stage, *Candidatus* Accumulibacter and *Zoogloea* increased in abundance, and later dominated the granular stage, together with *Defluviicoccus*, *Ferribacterium*, and *Rubrivivax*. These patterns of rapid initial changes followed by more gradual succession were also evident for the eukaryotic community. During the floccular stage, the microeukaryotic community was represented by members of the SAR supergroup, mainly *Stramenopiles* (*Amphifilaceae* and *Oomycota*) and *Alveolata* (*Sessilida*) superphyla (Supplementary Fig. 6). These were rapidly replaced by the *Roghostoma* lineage (*Rhizaria* superphylum) which decreased when granules started to dominate, with an accompanying transitional increase in abundance of rotifers. Finally, during the granular stage, ASVs affiliated to the *Rhogostoma* group kept their presence, dominating the eukaryotic community, accompanied by members of *Hacrobia* (*Cryptomonadales*), *Opisthokonta* (*Rotifera*) and *Alveolata* (*Sessilida*) superphyla (Supplementary Fig. 6).

We also evaluated eukaryotic communities through microscopical observations. Due to their dimensions and morphological characteristics, *Sessilida*, sessile peritrich ciliates, were the microeukaryotes most easily observed (Supplementary Fig. 1). They exhibit a sessile stage fixed by a stalk to a substrate as well as a dispersal stage with free-swimming forms that seek new substrates during their life cycles^31^. Even though high throughput sequencing did not allow the identification at lower taxonomic resolution of sessilids, microscopical observations allowed the distinction of some of its main families, such as *Epistylididae* or *Vorticellidae*. Species with morphology compatible with *Epistylis*, a colonial genus adapted to rapid water currents^32^, predominated throughout the study. Colonies with a varying number of zooids and stalk lengths (indicating a probable coexistence of different species within the genus) were distributed throughout the entire surface of the granules although some areas seem to be more favorable to the attachment than others. Cells with *Vortecellidae*-like morphology, having a stalk with spasmoneme, were also observed in the floccular stage, but the attachment of other sessile filter feeders seems to be inhibited during the process of granulation. During the intermediate and granular stages, small testate amebas of the genus *Rhogostoma* were also dominant (Fig. 2b). These protists are raptorial feeders^33^ with pseudopods which allow searching for bacterial prey that are loosely associated or permanently attached to surfaces.

### Parallel prokaryotic and eukaryotic community succession

**Fig. 3:**
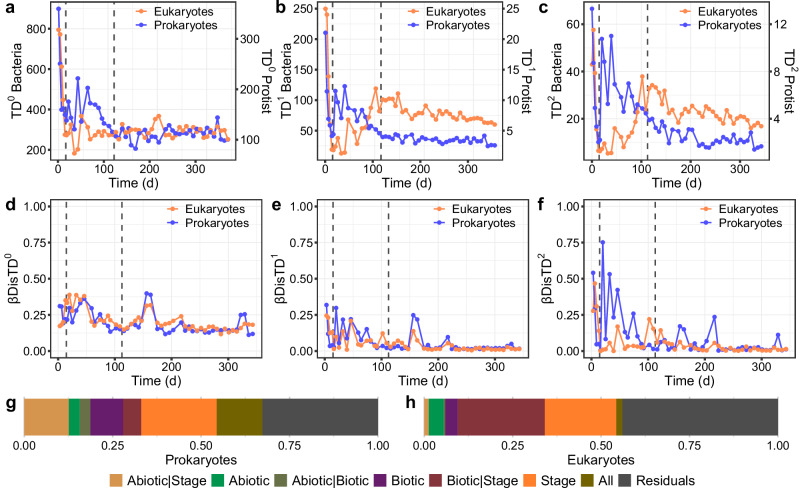
Microbial diversity and succession patterns. **a**–**c**, α-diversity (qTD) based on Hill numbers, as a function of order q. **d**–**f**, β-diversity (βDisTDq) between two successive sample points of the prokaryotic and eukaryotic communities. **a**, **d**, q of 0, relative abundance is not considered for the calculation. **b**, **e**, q of 1, relative abundance is considered. **c**, **f**, q of 2 more weight given to more abundant ASVs. Vertical dashed lines represent the three stages of biomass granulation in the bioreactor: Stage 1 – Flocs; Stage 2 – Intermediate; Stage 3 – Granules. Variance Partitioning Analysis describing the percentage of **g**, prokaryotic and **h**, eukaryotic community variation explained by sample data categories (Abiotic – sludge and performance parameters, Biotic – α-diversity of the other group, Stage – granulation stage of the reactor at each sample point). Variation not explained (residuals), shared variation explained by two categories (e.g., Abiotic | Biotic), and shared variation between the three categories (All) are also shown.

Both bacterial and eukaryotic communities suffered a similar drastic decrease in α-diversity during the first days of operation, with and without accounting for relative abundance (Fig. 3a–c). The taxonomic α-diversity of the bacterial community dropped by around 50% for ^0^TD and by over 60% for ^1^TD and ^2^TD, while the eukaryotic community showed even a higher loss, over 65% for ^0^TD and over 85% for ^1^TD and ^2^TD. At the beginning of the intermediate stage, the prokaryotic community increased its diversity, followed by a decreasing trend by the end of this stage and stabilization by the end of the experiment. The eukaryotic community increased in abundance by the end of the intermediate stage, followed by a decreasing trend throughout the granular stage. The loss of diversity over the experiment was more pronounced for the prokaryotic community than the eukaryotic. These trends were more pronounced when accounting for the dominant ASVs (i.e., when *q* = 2).

Prokaryotic and eukaryotic community succession patterns were parallel over the whole experiment (Fig. 3d, e), especially when accounting for rarer ASVs (i.e., *q* = 0 and *q* = 1; Supplementary Table 1). We observed fewer changes between successive communities at the end of the experiment, as reflected by taxonomic composition and α-diversity. In addition, the dominant prokaryotic community (*q* = 2) underwent more changes during the intermediate stage (Fig. 3f), in line with the taxonomic shifts observed (Fig. 2a). Both eukaryotic and prokaryotic communities presented parallel community succession, as revealed by constrained ordination and β-diversity correlation (Supplementary Fig. 3). Variance partitioning analysis revealed that biotic factors were more important for the eukaryotic community (Fig. 3h), whereas the prokaryotic community was more affected by abiotic factors such as nitrate or suspended solids concentration (Fig. 3g). The alpha diversity patterns of prokaryotic and eukaryotic communities explained relevant portions of the successional trends of the eukaryotic and prokaryotic communities, respectively (Fig. 3g, h). The prokaryotic succession was possibly affected by the increase in diversity of dominant eukaryotes by the end of the intermediate stage (Supplementary Fig. 3a). In the case of the eukaryotic succession, the higher prokaryotic diversity at the beginning of the experiment could have affected its succession patterns (Supplementary Fig. 3b).

### Network analysis reveals changes in community structure during granule formation

**Fig. 4:**
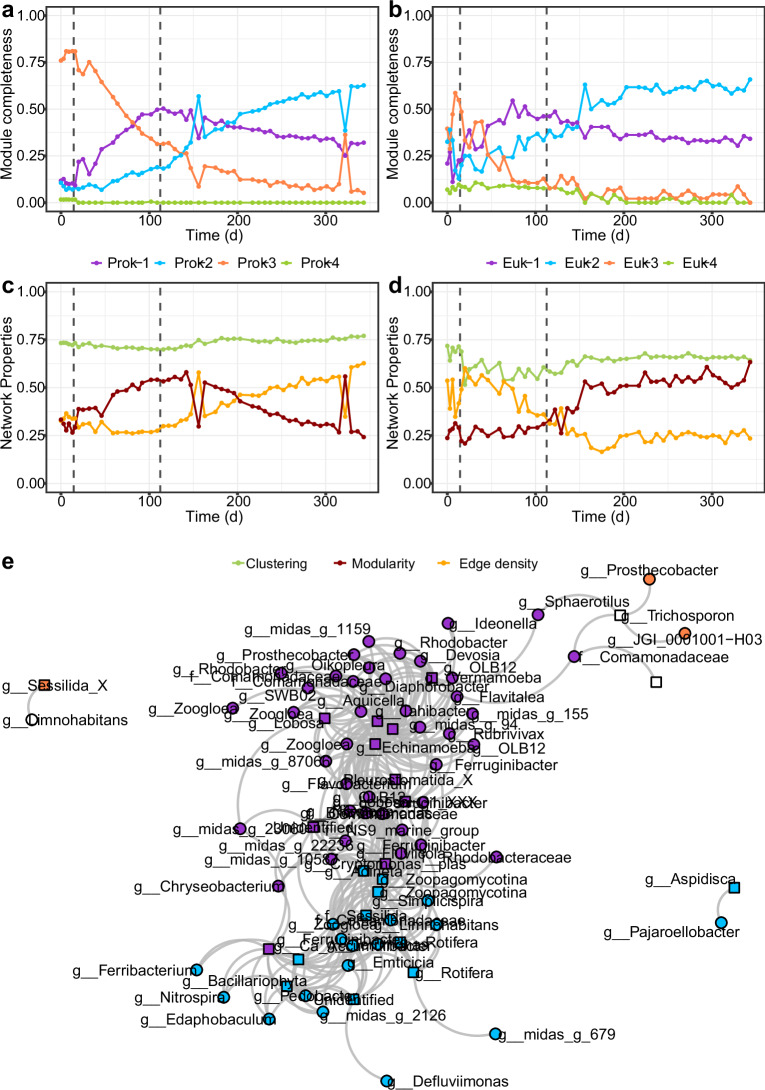
Composition and interaction structure dynamics of the microbial communities in the bioreactor. Community composition structure is measured as the proportion of nodes belonging to a given module (i.e., module completeness) of the **a**, prokaryotic and **b**, eukaryotic communities. Temporal patterns of modularity, clustering coefficient, and edge density of the **c**, prokaryotic and **d**, eukaryotic communities. **e**, Bipartite network showing the significant positive correlations between prokaryotic and eukaryotic core ASVs. Vertical dashed lines represent the three stages of biomass granulation in the bioreactor: Stage 1 – Flocs; Stage 2 – Intermediate; Stage 3 – Granules.

Both prokaryotic and eukaryotic networks were divided in four modules, or sub-communities, with parallel dynamics when attending to their proportion over time. Prok-3 and Euk-3 dominated the prokaryotic and eukaryotic communities in the initial floccular phase. Prok-3 was composed of several bacterial genera, most notably *Dechloromonas* and *Thauera*, whereas Euk-3 was composed mainly of members of the *Amphifilaceae* and *Sessilida* groups (Fig. 4a, b; Supplementary Fig. 4). These modules were replaced by Prok-1 and Euk-1 in the intermediate phase, where *Zoogloea* and members of the *Comamonadaceae* family dominate the prokaryotic module and members of the *Rotifera* and *Cryptomonadales* groups dominate the eukaryotic module (Fig. 4a, b; Supplementary Fig. 4). Subsequently, Prok-2 and Euk-2, dominated in the granular phase (Fig. 4a, b; Supplementary Fig. 4). These modules were composed mainly of *Candidatus* Accumulibacter and *Zoogloea*, and members of the *Sessilida* and *Rotifera* groups, respectively (Supplementary Fig. 4). Time-point network properties revealed differences between prokaryotic and eukaryotic dynamics of community structure (Fig. 4c, d), although both communities presented less clustering during the intermediate stage. Modularity and edge density followed opposite trends in both communities. In the prokaryotic community, modularity increased during the intermediate stage and decreased during the granular stage. The eukaryotic modularity was at a minimum during the first granulation stages and increased starting the granulation stage.

We further investigated the main potential microeukaryote-prokaryote interactions in the reactor using bipartite networks. Thus, we calculated the eukaryote-prokaryote correlation networks keeping only the nodes that were detectable through most of the experiment (>75% samples), as we aimed to evaluate inter-kingdom associations across the members of the community that thrived in different granulation contexts, constituting the core community. Besides, we used the module membership of the previously calculated networks to assess the potential interactions between sub-communities with similar behavior (Fig. 4e). In this network, the nodes closely connected belonged to the prokaryotic and eukaryotic modules behaving similarly (i.e., nodes from Prok-1 and Euk-1, or nodes from Prok-2 and Euk-2). Throughout the granulation stage, Prok-2 and Euk-2 sub-communities dominated the microbial communities, with an abundance of 55.3% and 29.1% by the end of the experiment. However, they were simpler, with fewer nodes and connections than Prok-1 and Euk-1, which were the dominant communities during the intermediate stage (Fig. 4, Supplementary Table 2). The most abundant ASVs from Prok-2 belonged to *Candidatus* Accumulibacter (30.4%), *Ferribacterium* (9.9%), and *Zoogloea* (9.8%) genera, and from Euk-2 to the *Sessilida* (21.0%) and the *Rotifera* (5.4%) groups (Supplementary Fig. 4).

### Prokaryotic and eukaryotic community succession is governed by different ecological processes

**Fig. 5:**
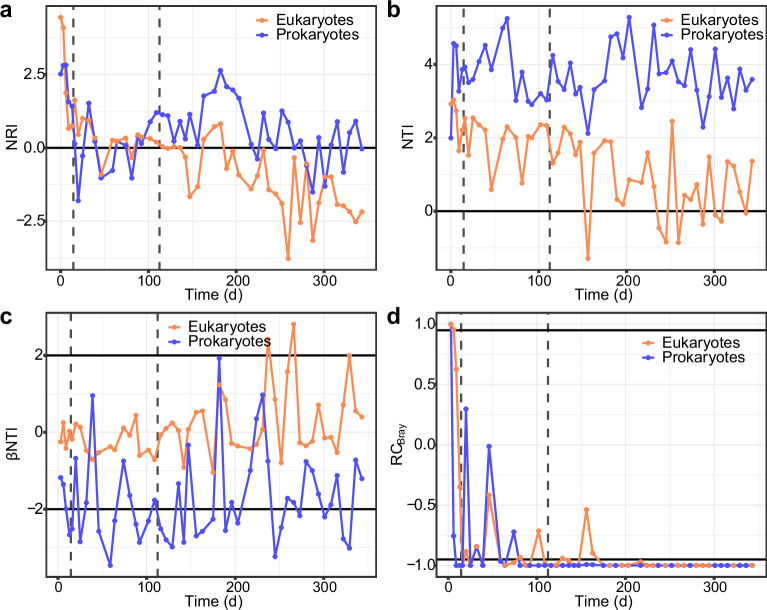
Temporal changes in phylogenetic structure and successional turnover in the bioreactor. Community phylogenetic structure is assessed via **a** net relatedness index (NRI) and **b** nearest taxon index (NTI). Null model analysis results between two successive sample points assessed by **c**, phylogenetic turnover based on βNTI, and by **d**, taxonomic turnover based on RCBray. Horizontal lines indicate thresholds for significant deviations from the null expectation. Vertical dashed lines represent the three stages of biomass granulation in the bioreactor: Stage 1 – Flocs; Stage 2 – Intermediate; Stage 3 – Granules.

We assessed the phylogenetic alpha dispersion of the microbial communities over time by calculating the net relatedness index (NRI) and the nearest taxon index (NTI), which examines the clustering/dispersion of phylotypes (Fig. 5a, b). First, to justify the use of null models on phylogenetic α and β diversities, we verified the phylogenetic signal across relative short phylogenetic distances (Supplementary Fig. 5). We observed that the NRI (prokaryote: 0.59 ± 1.10; eukaryote: -0.36 ± 1.55) values were lower than the NTI (prokaryote: 3.69 ± 0.76; eukaryote: 1.30 ± 1.08) values in both communities revealing that deterministic assemblage is more relevant at terminal levels in the phylogeny (e.g., genus/species level rather than phylum/broader groups). Besides, we found that the NRI values were much higher in the first samples, and then decreased. In the prokaryotic community the higher NRI values match with the floccular stage (NRI = 2.23 ± 0.68), and then was at some extent stable around 0 (0.41 ± 0.99). The NRI values of the eukaryotic community decreased faster during the first samples, reaching values close to 0 in the intermediate stage and negative values in the granular stage. This decreasing trend is also observed in the eukaryotic NTI, reaching values close to 0 at the end of the experiment. The NTI of the prokaryotic community, however, remained positive during the whole experiment.

Then, we evaluated the changes in β-diversity using the RC_bray_ (based on taxonomic turnover), and βNTI (based on phylogenetic turnover) metrics (Fig. 5c-d). The RC_bray_ values were higher than the null expectation (RC_bray_ > 0.95), then the values decreased in both communities, however, their dynamics differed. In both, the prokaryotic and eukaryotic communities, RC_bray_ values were within the null expectation (|RC_bra_|< 0.95) in the initial and intermediate granulation stages, and in the granular stage were overall lower than expected (RC_bray_ < -0.95). The βNTI dynamics also differed between prokaryotic and eukaryotic communities. The prokaryotic community had overall negative βNTI values, mostly lower than the null expectation (βNTI<-2). However, the eukaryotic community had βNTI values around 0, and by the end of the experiment, some values were higher than the null expectation (βNTI>2).

## Discussion

To date, there is still a lack of comprehensive studies addressing the microbial associations and their ecological implications occurring during granule granulation in SBRs, especially those involving inter-kingdom interactions, as relatively few studies have been conducted on the role of eukaryotes on the granular sludge process. Sludge granulation has been described to occur in several steps^4,34^, and indeed, here, we identified three ecological stages, floccular, intermediate, and granular, when both the microbial community and sludge parameters changed.

The initial stages of the granulation process are controlled by different environmental factors and properties of the biomass^34,35^, which are closely related to the microorganisms inhabiting and dominating the bioreactors. We found the prokaryotic community dominated by bacteria (e.g., *Acinetobacter* sp., *Dechloromonas* sp., or *Thauera* sp., Fig. 2a) which have been described as early biofilm colonizers and important extracellular polymeric substances (EPS) producers^18,36,37^ and commonly detected during the initial phases of granulation^3,28,38^. A diverse community of microeukaryotes was also present, mostly protozoa with different feeding modes and motility which promote microbial activity and aggregation against predation^19,39^. *Rhogostoma* increased its abundance, dominating the community by the end of the floccular stage (Fig. 2b). They have been observed as dominating eukaryotic communities in several WWTPs^40–43^ and found to be represented by a single species, *Rhogostoma minus*, which recently have received researchers attention not only for its widespread distribution but for hosting well-known human pathogenic *Legionellales*^44^. The abundance of the *Sessilida* group (*Alveolata* supergroup) in turn reached a minimum by the end of the floccular stage. This could be explained by a sudden increase of rotifer populations, that could be ingesting *Sessilida* individuals^45^. On the other hand, it may be related to the decrease of available preys^46^ as a result of the washing out of the non-granulated microorganisms, or by the species filtering occurring in the reactor due to the acclimation of the sludge community to the new environmental conditions. This is consistent with the pronounced drop in microbial α-diversity, with and without accounting for relative abundance, and the community successional patterns, indicating a higher turnover in community composition during the first stages of granulation, especially when accounting for the relative abundance (Fig. 3). The switch from complex to simple and easily biodegradable substrate (i.e., acetate) could emphasize the drop in prokaryotic α-diversity and the higher community dynamics contributing the strong selective processes exerted by reactor dynamics during the first stage of granulation^28,38,47^. Consequently, the changes in the prokaryotic community structure would also influence the diversity and abundance of heterotrophic eukaryotes^14,48^. In addition, the reactor wash-out dynamics would also induce the reduction of eukaryotic diversity, especially free-swimming ciliates^39^. Indeed, module completeness revealed the division of prokaryotic and eukaryotic communities into sub-communities (Fig. 4) with, presumably, different environmental preferences^49^. During the floccular stage both were dominated by a disappearing sub-community (Prok-3, Euk-3), allegedly adapted to the initial activated sludge conditions. Prokaryotic and eukaryotic initial communities had high NRI and NTI values that decreased during granulation (Fig. 5) revealing phylogenetic clustering, previously observed in activated sludge systems^49^, which could be indicative of the deterministic forces (such as the high wash-out dynamics and the new environmental conditions described) driving community assembly. These selective forces would affect mainly narrower taxonomic groups (e.g., genus taxonomic level rather than order or phylum), as revealed by NRI values close to 0, and overall positive NTI values.

During sludge granulation, the suspended biofilms further develop and increase in diameter where oxygen and substrate gradients are created within the granule, providing new niches to be colonized. Indeed, when granules started to emerge during the intermediate stage, the microbial community displayed high fluctuations. During this stage, important EPS producers such as *Zoogloea* sp.^50^ and others associated with the production of a resistant matrix of structural EPS like *Ca*. Accumulibacter^51^ substantially increased in abundance (Fig. 2a). Members of the *Rhogostoma* protistan group decreased in relative abundance in favor of other micro-eukaryotes within the *Cryptomonadales*, *Rotifera* and *Sessilida* groups (Fig. 2b). The areas with greater abundance of peritrichs (*Sessilida*) were those with irregularities and grooves as they can find protection against water turbulence and hence, fewer possibilities of getting detached^52^ although signals of abrasion, i.e. lack of zooids, were frequently observed. The denser peritrichous colonization during this stage (Supplementary Fig. 1) may reflect the increased availability of settlement sites and the favorable conditions for the development of these bacterivore ciliate communities^53,54^. Additionally, the flourish of stalked ciliates could have improved the granulation process serving as the backbone for biofilm development^17^.

The prokaryotic community stabilized at the beginning of the granular stage, resulting in a replacement of the initial community by a simpler prokaryotic community dominated by a handful of bacterial genera including *Ca*. Accumulibacter, *Defluviicoccus*, *Ferribacterium*, *Rubrivivax*, and *Zoogloea* (Fig. 2a), which are commonly detected in reactors performing simultaneous biological removal of organics, nitrogen and phosphorous in wastewater treatment plants^55–57^. We indeed observed nitrification and phosphorous removal to improve during this phase (Supplementary Fig. 2). In agreement with the progression of the granulation, the biomass concentration in the reactors was doubled. The granular size increase changed the microenvironment within the granule matrix, contributing to the deterministic factors influencing the community assembly process during this stage, both when considering the terminal levels in the phylogeny (NTI), or the taxonomic turnover between successive samples (RC_Bray_). The phylogenetic turnover between successive prokaryotic communities (βNTI) also indicated the importance of deterministic factors driving community succession during the intermediate and the first half or granular stages, around day 200 (Fig. 5). This agrees with the observed higher influence exerted by abiotic factors, such as nitrate concentration in the prokaryotic community (Fig. 3g). However, the importance of stochastic factors increased during the granular stage, evidenced by the higher proportion of NRI and βNTI values within the null expectation after day 200 (Fig. 5c). The overall lower values of NRI and NTI and overall random phylogenetic turnover, compared to prokaryotic communities (Fig. 3h, Fig. 5), are consistent with the eukaryotic community succession being more affected by biotic factors, such as random inter-kingdom interactions, competition, predation and mutualism^58^ or other random factors such as available settlement sites for reproduction. In addition to trophic interactions, eukaryotic and prokaryotic communities also compete for physical niches. For example, due to their similar growth pattern filamentous bacteria compete for settling sites with peritrichous ciliates^59^, which could contribute to the opposite trends in prokaryotic and eukaryotic richness during early granule formation (Fig. 3a–c).

Network analysis also evidenced the community successional patterns associated with increased granule size. A higher taxonomic turnover was revealed by emerging sub-communities (Prok-1, Euk-1; Supplementary Fig. 4). The presence of differently sized aggregates would promote the generation of different niches for functional groups^60^, like the dominating Prok-1 sub-community (members of the *Comamonadaceae* family and *Zoogloea* sp.). This niche differentiation process could also be revealed by the initial increase in prokaryotic modularity coupled with a decrease in clustering coefficient^61^. The eukaryotic community presented a similar increasing modularity trend by the end of the intermediate stage, when peritrichs of the *Sessilida* group and numerous *Rotifera*, within the Euk-2 sub-community, emerged (Supplementary Fig. 4). Besides, this stage was also characterized by a stabilization of a *Cryptomonas* population (from Euk-1, Supplementary Table 2), which would not compete with the Euk-2 sub-community for settling sites^62^. *Cryptomonas* is a mixotrophic genus with species that can combine photosynthetic activity with utilization of exogenous carbon sources, here, uptake of supplemented acetate or/and engulfment of bacteria to maintain or enhance their growth, although fully heterotrophic conditions will not allow their survival^63^. The abundance of *Cryptomonas* could be also related to nitrogen metabolism. In an experiment performed by Krustok et al.^64^ in municipal wastewater treating photobioreactors, this flagellate was able to grow to a higher concentration with nitrogen existing mostly as NH_4_-N.

Hence, contrary to the prokaryotic community, the decrease in edge density suggests that the eukaryotic community turned simpler, also evidenced by the observed drop in α-diversity, and more niche-specialized during the granular stage (Fig. 3, Fig. 4cd)^65^. This simplification was also observed in the fewer correlations between the Euk-2 and Prok-2 sub-communities in the bipartite network (79) compared with the Euk-1 and Prok-1 (178). The specialization of the eukaryotic community is reflected by the increasing abundance of *Rotifera* and *Sessilida* groups adapted to granules which provide the space where they can attach avoiding the washing out of the system^17,45^. Both, being filter feeders, create water currents and ingest suspended prey and fine sludge particles more efficiently removing non-flocculated bacteria^19,45^.

Despite the long settling times used in the reactor, and thus applying a low wash-out regime to the biomass, granules started to emerge after 16 days and at day 112 they were fully developed. Washing out the non-granulated biomass is considered an important selection force for sludge granulation. However according to the results presented here, high wash-out rates are not a prerequisite for granulation to occur, although the process is accelerated considerably. By way of comparison, in a previous experiment using the same reactor set-up, but with a settling time of 2 minutes, granulated biomass dominated the reactor already after 25 days^28^. Granulation at low wash-out dynamics has also been reported by other researchers^66–68^, even with a total retention of biomass in the reactor^69^. However, when long settling times are applied, higher shear forces have been found necessary to achieve granulation^26,70^. These results suggest that other factors than short settling time may be more important for granulation, such as high hydrodynamic shear forces and feast-famine regimes. This opens the door to explore alternative strategies for granulation in different conditions, such as continuous operation^71^.

Altogether, our findings provide insights in the successional patterns of micro-eukaryotes during granule formation and the interkingdom interactions of this population with the prokaryotic community. Here, deterministic forces were important during the initial stages of sludge granulation, presumably caused by the acclimation of the microbial community to new environmental factors. Changes in the prokaryotic community structure determined the successional patterns of the micro-eukaryotic communities. Although inter-kingdom interactions were shown to affect community succession during the whole experiment, during granule development random factors like the availability of settlement sites or drift acquired increasing importance.

## Methods

### Reactor set-up and operational conditions

The SBR was inoculated with activated sludge from the Hammargården wastewater treatment plant designed for biological nitrogen and phosphorus removal (Kungsbacka, Sweden) and operated at a settling time of 30 min for 343 days. The SBR, previously described in detail^28^, had a working volume of 3 L. Synthetic wastewater was used and consisted of 994.2 mg L^−1^ NaCH_3_COO, 443.8 mg L^−1^ NH_4_Cl, 139.5 mg L^−1^ K_2_HPO_4_, 56.5 mg L^−1^ KH_2_PO_4_, 12.5 mg L^−1^ MgSO_4_·7H_2_O, 15.0 mg L^−1^ CaCl_2_, 10.0 mg L^−1^ FeSO_4_·7H_2_O, and 1 mL L^−1^ micronutrient solution^28^. The feed had an organic loading rate of 2 kg COD m^−3^d^−1^, N-load of 0.3 kg NH_4_-N m^−3^d^−1^ and P-load of 0.1 kg PO_4_-P m^−3^d^−1^ resulting in a COD:N:P ratio of 20:3:1. The reactor was operated at room temperature (20–22 ˚C) with a volumetric exchange ratio of 43%, in a 4-hour cycle of 5 min filling, 55 min anaerobic/anoxic phase, 143 min aerobic phase, 30 min settling, 5 min withdrawal and 2 min idle phase.

### Analytical methods

Effluent samples were collected and filtered (0.2 µm pore size), DOC and total nitrogen (TN) were measured with a TOC-TN analyser (TOC-V, Shimadzu, Japan), and acetate, ammonium, nitrite, nitrate, and phosphorus were measured using a Dionex ICS-900 ion chromatography. Total suspended solids and volatile suspended solids in the reactor and in the effluent were measured according to standard methods^72^. Microscopy was performed using an Olympus BX60 light microscope (Olympus Sverige AB, Solna, Sweden) and particle size was assessed with ImageJ^73^. A cycle study was performed on day 99 using a flexible plastic tube (ø 1 cm) attached to a syringe to sample the reactor at different heights during the aerobic phase and in the upper third of the sludge bed during the anoxic phase, to obtain representative samples.

### DNA extraction, amplification, and sequencing

A total of 52 samples were collected for DNA analysis, used for both prokaryote and eukaryote amplicon sequencing analysis. DNA was extracted using the DNeasy PowerSoil Kit (Qiagen) following manufacturer’s instructions. The rDNA libraries were constructed as described in Liébana et al.^28^. Shortly, for prokaryotes, the V4 region of the 16S rRNA gene was amplified using the forward primer 515’F (5´-GTGBCAGCMGCCGCGGTAA-3´) and the reverse primer 806R (5´-GGACTACHVGGGTWTCTAAT-3´), indexed according to Kozich et al.^74^. For eukaryotes, the V9 region of the 18S rRNA gene was amplified using the 1391f (5´-GTACACACCGCCCGTC-3´) forward primer and the EukBr (5´-TGATCCTTCTGCAGGTTCACCTAC-3´) reverse primer^75^, indexed according to Vences et al.^76^. The PCR products were sequenced with a MiSeq (Illumina) using the reagent kit v3 (PE 2×300) and v2 (PE 2×150) for the prokaryotic and eukaryotic libraries respectively.

### Sequence processing

Sequence reads were processed using the *DADA2* R version 1.22 package^77^ and *USEARCH* version 11^78^, as previously described^79^. The obtained count tables were used to generate consensus tables consisting of ASVs detected using both pipelines with the function *subset.consensus* implemented in *qdiv* (https://github.com/omvatten/qdiv). The taxonomic assignment was performed using the SINTAX algorithm^80^ based on the MiDAS database v.4.8.1^81^ for 16S reads and PR2 v.4.14 database^82^ for 18S reads. We used the MiDAS database because it covers the global diversity of microbes in wastewater treatment systems^83^; and the PR2 database was chosen because it consists of a comprehensive-curated database that places eukaryotic sequences within a coherent ranked taxonomic framework covering eukaryotic, mainly protistan, diversity^82^. The datasets were rarefied, subsampling each sample to 43329 and 31420 reads for the prokaryotic and eukaryotic count tables, respectively. Sequences were aligned with the *msa* R package^84^ and a maximum likelihood tree was generated using *phangorn* R package^85^ using a GTR + GI model. Taxonomic α-diversity was calculated using Hill numbers^86^ with the *hillR* R package^87^. Hill numbers, also called effective numbers, are a set of diversity indices that uses diversity order (q) to determine the weight given to the relative abundance of each ASV^88^. When q is 0, the relative abundance is not considered, and so, this value represents the richness. When q is 1, ASVs are weighted exactly according to their relative abundance, this value would equal the exponential Shannon index (exp(H)). Finally, when q is 2, more weight is given to abundant ASVs, representing the reciprocal Simpson index (1/D)^88^. The effect on α-diversity of biological and environmental parameters was evaluated using linear models. The Hill numbers framework was also used to calculate β-diversity^79^, dissimilarity indices (^q^βdis) constrained between 0 and 1 using *qdiv*. Community succession and its relationship with environmental parameters were evaluated by performing distance-based Redundancy Analysis (dbRDA) and variance partitioning analysis using Bray-Curtis dissimilarity with the *vegan* R package^89^. For these analyses, we defined three categories: biotic, abiotic, and stage. Biotic factors correspond to the α-diversity values eukaryotic communities for prokaryotic succession and vice versa, abiotic factors correspond to the reactor parameters measured (described in section 4.2), and the stage corresponds to the granulation stage defined in this work. Before performing variance partitioning, we conducted a permutation test in constrained ordination to choose the best fitting model using the *ordistep* function in the *vegan* R package, which resulted in the selection of nitrate and suspended solids concentration as the abiotic factors selected to model prokaryotic community succession, and phosphate and total organic carbon for eukaryotic succession.

### Network analysis

Network analysis was conducted to evaluate the interaction patterns of the bacterial and eukaryotic communities. We first removed the ASVs present in less than 10% of samples and an abundance lower than 0.1% (resulting in 411 and 125 ASVs remaining in the prokaryotic and eukaryotic datasets respectively). Then, we calculated every potential co-occurrence between the ASVs applying two correlation models, Spearman’s rank correlation and Sparse Correlations for Compositional data (SparCC), implemented in the *SpiecEasi* R package^90^. Co-occurrence were considered when the Spearman’s correlation coefficient (ρ) and SparCC R-corr absolute values were higher than 0.6, and their false discovery rate (FDR) corrected *p*-values lower than 0.05. The resulting networks consisted of 325/67 nodes and 6010/308 edges for the prokaryotic and eukaryotic communities, respectively. Co-occurrence patterns of the core communities and the potential interkingdom associations were assessed on filtered networks, keeping the nodes present in more than 75% of samples. Network visualization was performed with the *igraph* R package^91^ and nodes’ module membership calculation was calculated with the cluster walktrap algorithm in the *igraph* package to find the minimal amount of densely connected subgraphs (sub-communities). We also calculated the proportion of ASVs (module completeness) and the abundance of each assigned module in the networks. In addition, we applied the method developed by Ortiz-Álvarez et al.^61^ to calculate the individual co-occurrence networks of each time-step sample, assessing their individual properties and the microbial communities structure over time.

### Microbial community phylogenetic dispersion against a null expectation

We assessed the influence of stochastic and deterministic processes in the community succession by means of null model analysis on the within (α) and between (β) sample phylogenetic diversity, coupled with taxonomic turnover^92^. Prior applying this framework we tested the phylogenetic signal, that is, if closely related ASVs have similar environmental preferences^93^, using a Mantel correlogram between ASV environmental optima and their phylogenetic distance. The environmental optima of each ASV were calculated as the abundance-weighted mean of each environmental parameter. Then, we calculated the pairwise ASV phylogenetic distance using the branch lengths of the phylogenetic tree previously calculated, using the cophenetic function of the *ape* R package^94^.

The phylogenetic α-diversity structure was studied calculating the net relatedness index (NRI) and the nearest taxon index (NTI), using the ses.mpd and ses.mntd functions (null.model = “taxa.labels”, abundance.weighted = TRUE) of the *picante* R package^95^. These indices correspond to the standardized effect size of the mean pairwise diversity (MPD) and the mean nearest taxon distance (MNTD), respectively. The NRI measures the dispersion across the phylogeny, and the NTI measures the dispersion of closely related taxa^96^. The closer they get to zero, the closer the phylogenetic structure of the community is to the null expectation, reflecting the higher influence of stochasticity. Values below zero describe phylogenetic overdispersion, and above zero phylogenetic clustering, both caused by deterministic processes^97^.

Null models applied to phylogenetic β-diversity were used to study whether phylogenetic turnover across two samples was more, or less, similar than that expected by chance. For this, the β-Nearest Taxon Index (βNTI) was calculated with the *qdiv* package^79^, which measures if the phylogenetic turnover is different than the null expectation. The β mean nearest-taxon distance (βMNTD) measures the mean phylogenetic distance between the most closely related ASVs in two communities, and was first calculated based on relative abundance data^98^. The null distribution of the βMNTD is provided by shuffling the ASVs across the tips of the phylogenetic tree in 999 iterations and using the resulting phylogenetic relationships to calculate the βMNTD_null_. The resulting βNTI values reflect the distance of the phylogenetic turnover between two communities to a null expectation. Values close to zero, close to the null expectation, indicate the higher effect of stochasticity shaping the community assembly, while values of |βNTI|> 2 are considered to indicate that the observed turnover is significantly deterministic^99^.

Taxonomic turnover was assessed using Raup-Crick based measures, calculated using the *qdiv* package, which quantify the deviation of the observed turnover from that expected if the community was randomly assembled. For this, we compared the observed Bray-Curtis dissimilarity with a null distribution, and the deviation between the observed Bray-Curtis and the null distribution is standardized to vary between −1 and +1^100^. To create the null distribution, the total number of ASVs and read counts of each sample were kept constant, but the identity and distribution of the ASVs were randomized in 999 iterations. |RCbray| values > 0.95 are considered to reveal that the observed community composition is different from the null expectation, whereas |RCbray| values < 0.95 are consistent with the effect of drift^98^.

## Supplementary information

Supplementary material

Supplementary Table S2

## Data Availability

Raw sequence reads are deposited at the European Nucleotide Archive (ENA) repository under the project code PRJEB71975. The code and the necessary data to reproduce all the analyses are included in a Figshare repository (https://figshare.com/s/dfd2d3546e719829fad9, will be available upon acceptance).
