# Supplementary material for: Community successional patterns and inter-kingdom interactions during granular biofilm development
Source: NPJ Biofilms Microbiomes. 2024 Oct 20;10:109. doi: 10.1038/s41522-024-00581-x (PMC11490564; doi:10.1038/s41522-024-00581-x)
Supplement: Supplementary file 1 — Supplementary material [file 41522_2024_581_MOESM1_ESM.pdf]

SUPPLEMENTARY INFORMATION

Community successional patterns and inter-kingdom interactions during granular biofilm development.

Miguel de Celis[12#], Oskar Modin[3], Lucía Arregui[1], Frank Persson[3], Antonio Santos[1], Ignacio Belda[1], Britt-Marie Wilén[3#], and Raquel Liébana[3*#],

[1] Department of Genetics, Physiology and Microbiology, Microbiology Unit, Faculty of Biological Sciences, Complutense University of Madrid, Spain

[2] Instituto de Ciencias Agrarias; Consejo Superior de Investigaciones Científicas, Madrid, Spain

[3] Division of Water Environment Technology, Department of Architecture and Civil Engineering, Chalmers University of Technology, Gothenburg, Sweden

[*] Current address: AZTI, Marine Research Division, Basque Research Technology Alliance (BRTA), Sukarrieta, Spain

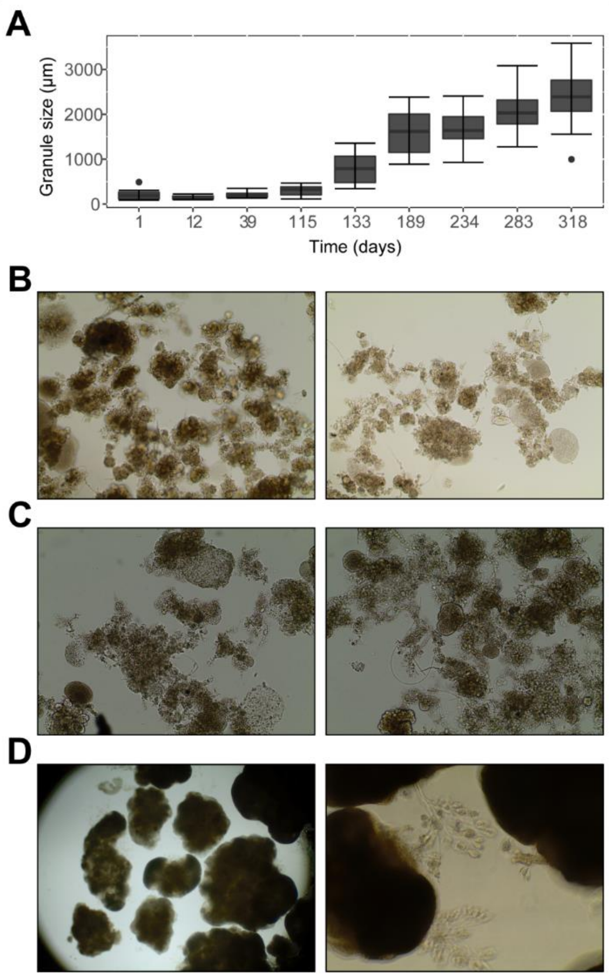

Supplementary Figure 1. Particle size of the reactor sludge during experiment. A, particle size (µm) of granules (n=10). Microscopic image of sludge during the B, floccular; C, intermediate; and D, granular stage.

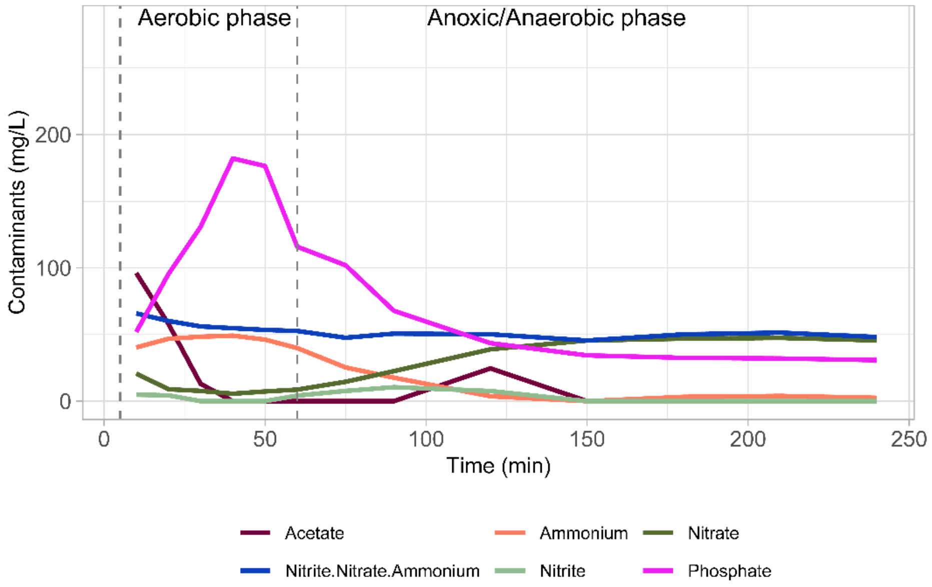

Supplementary Figure 2. Cycle study results for acetate-C, nitrate-N, nitrite-N, ammonium-N, phosphate-P and total nitrogen expressed as the sum of nitrate, nitrite and ammonium.

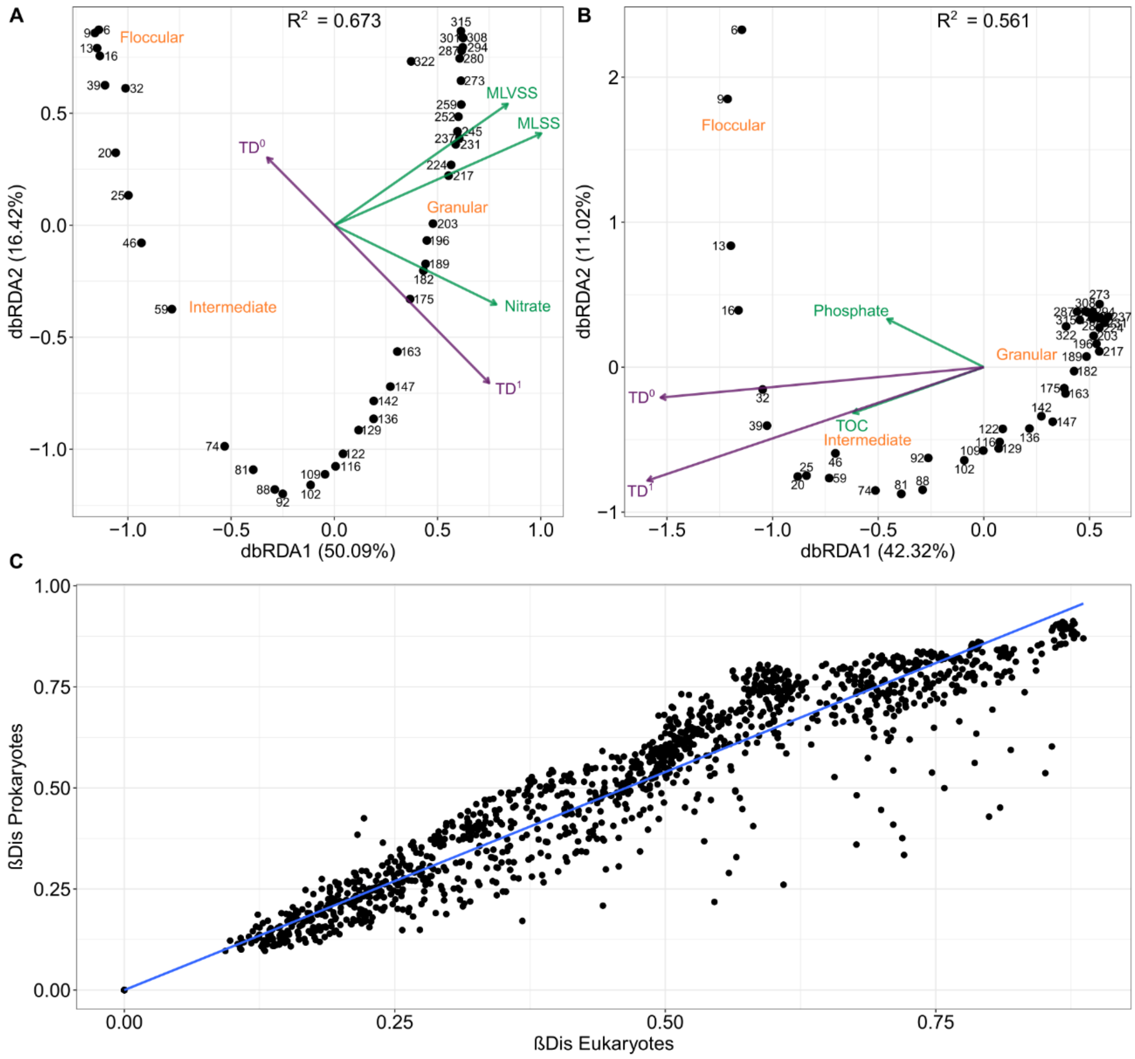

Supplementary Figure 3. Constrained principal coordinates analysis (dbRDA) based on Bray-Curtis dissimilarity of square root transformed count tables. A, Prokaryotes. B, Eukaryotes. Significant variables ($p < 0.05$) were selected with an automated stepwise model (ordistep). Explained variance of each axis are indicated in parenthesis. The numbers refer to days of reactor operation. C, Correlation between prokaryotic and eukaryotic β-diversity, as measured by Bray-Curtis dissimilarity.

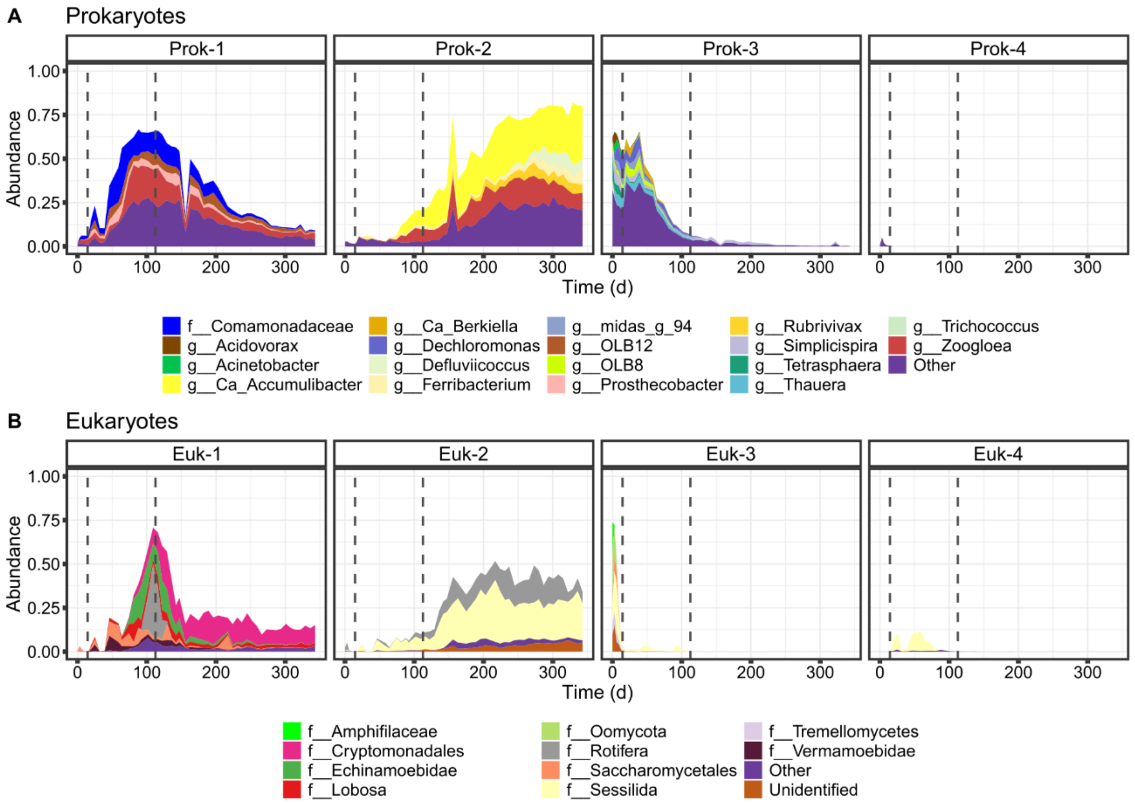

Supplementary Figure 4. Taxonomic profiling of the modules detected in the microbial communities. A, taxonomic composition at the genus level of the modules detected in the prokaryotic co-occurrence network. B, taxonomic composition at the family level of the modules detected in the eukaryotic co-occurrence network. “Other”, includes genera with less than 5% abundance in at least one sample point; and “Unidentified”, taxonomically unassigned taxa.

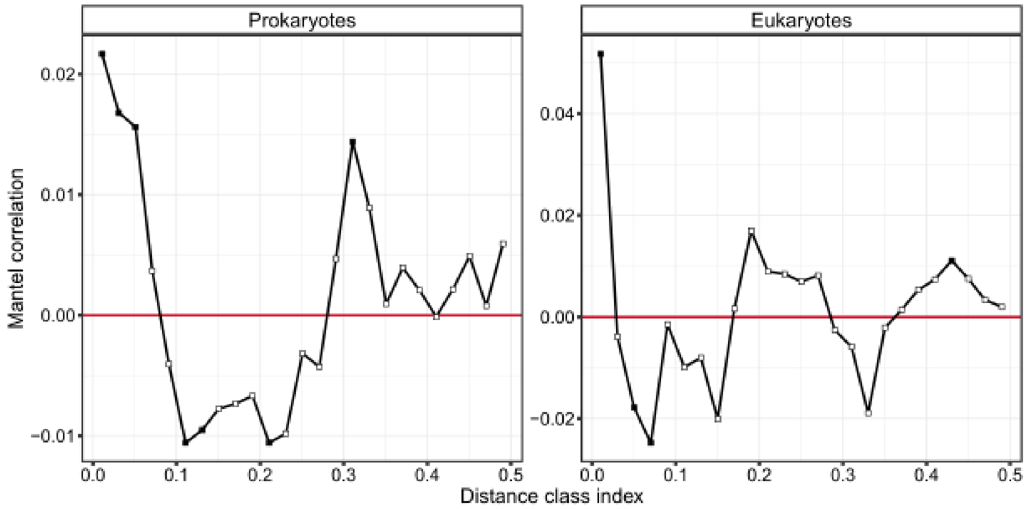

Supplementary Figure 5. Mantel correlograms showing phylogenetic signal. Pearson correlation resulting from Mantel correlogram (999 permutations) between the ASV environmental (accounting for operational conditions and granulation stage) and phylogenetic distances for A, prokaryotic and B, eukaryotic communities. Significant correlations (solid squares) at low distances indicate phylogenetic signal in species ecological niches.

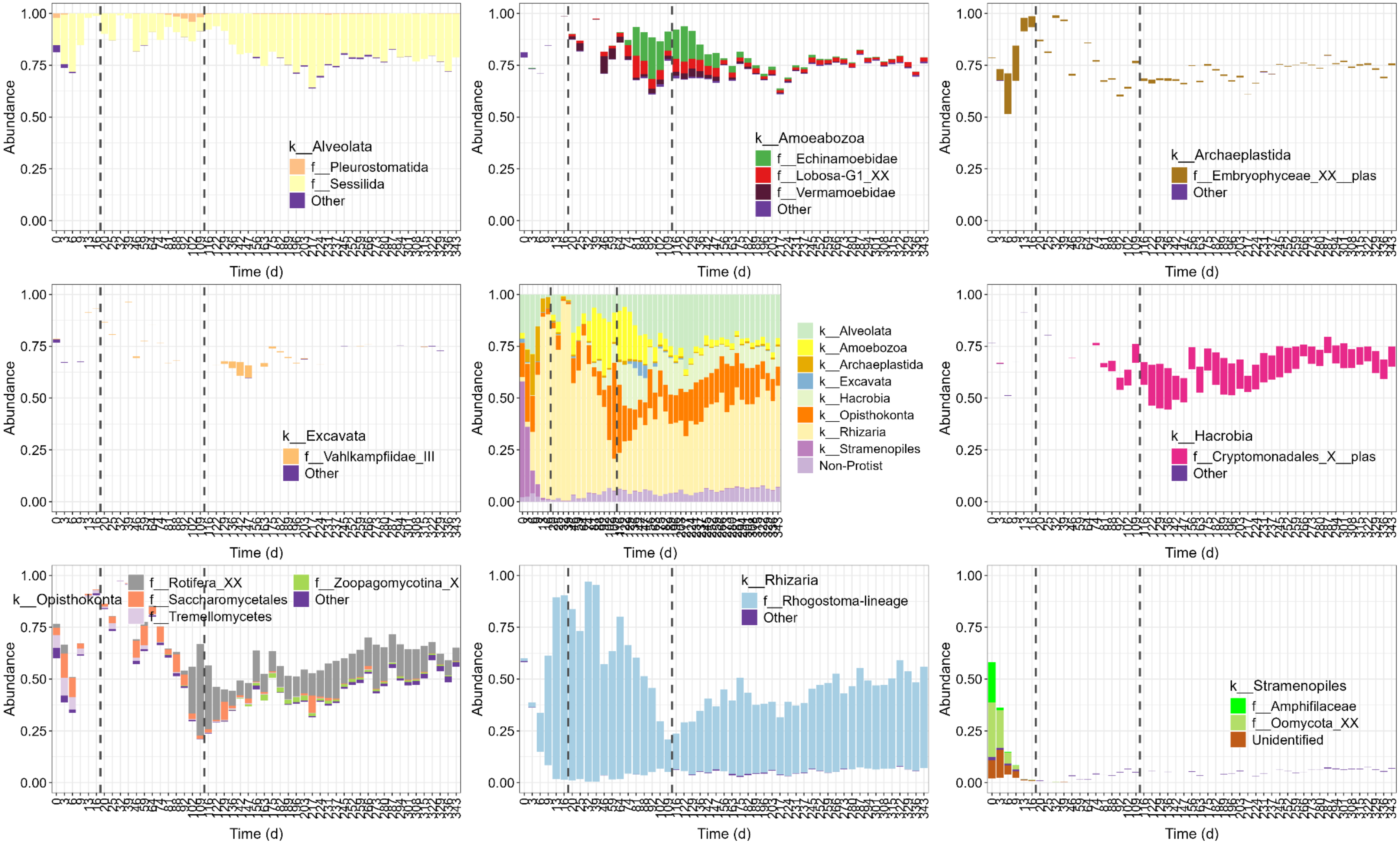

k__Alveolata
f__Pleurostomatida
f__Sessilida
Other
k__Amoeabozoa
f__Echinamoebidae
f__Lobosa-G1_XX
f__Vermamoebidae
Other
k__Archaeplastida
f__Embryophyceae_XX__plas
Other
k__Excavata
f__Vahlkampfiidae_III
Other
k__Alveolata
k__Amoebozoa
k__Archaeplastida
k__Excavata
k__Hacrobia
k__Opisthokonta
k__Rhizaria
k__Stramenopiles
Non-Protist
k__Hacrobia
f__Cryptomonadales_X__plas
Other
k__Opisthokonta
f__Rotifera_XX
f__Saccharomycetales
f__Tremellomycetes
f__Zoopagomycotina_X
Other
k__Rhizaria
f__Rhogostoma-lineage
Other
k__Stramenopiles
f__Amphifilaceae
f__Oomycota_XX
Unidentified
Abundance
Time (d)

Supplementary Figure 6. Temporal dynamics of eukaryotic taxonomic composition. Taxonomic distribution of the eukaryotic community at the superphylum and family (or equivalent according to PR2 database) levels. The middle panel represent the taxonomic profiling at the superphylum level and each panel next to it represent the taxonomic profiling at the family level of each superphylum.

$\beta DisTD^0$	$\beta DisTD^1$	$\beta DisTD^2$
0.595 ***	0.668 ***	0.394 **

Supplementary Table 1: Spearman's rank correlations between prokaryotic and eukaryotic community succession, taxonomic β-diversity between successive sample points, at diversity order of q.

Asterisks denote the significance levels (***p-value < 0.001, **p-value < 0.01 and *p-value < 0.05).

Supplementary Table 2: Taxonomic and abundance information of the nodes from the core bipartite network.

Provided as a separate spreadsheet.
